# Evaluation of Self-Propelled High-Energy Ultrasonic Atomizer on Azoxystrobin and Tebuconazole Application in Sunlit Greenhouse Tomatoes

**DOI:** 10.3390/ijerph15061088

**Published:** 2018-05-28

**Authors:** Yan-Jie Li, Yi-Fan Li, Rong-Hua Chen, Xue-Sheng Li, Can-Ping Pan, Jian-Li Song

**Affiliations:** 1 Department of Applied Chemistry, College of Science, China Agricultural University, Beijing 100193, China; yanjieli0913@outlook.com; 2 Department of Chemistry and Biochemistry, University of California San Diego, La Jolla, CA 92093, USA; yil909@ucsd.edu; 3 Institute of Pesticide and Environmental Toxicology, Guangxi University, Nanning 530005, China; ronghuachen5533@163.com (R.-H.C.); Lixueshe@msu.edu (X.-S.L.)

**Keywords:** self-propelled ultrasonic atomizer, pesticide residue, tomato, sunlit greenhouse

## Abstract

In this study, a self-propelled high-energy ultrasonic atomizer was evaluated in terms of deposition on the canopy, the loss to the ground, and fungicide residues in cherry tomato and tomato. Artificial collectors fixed to the upper side and underside of the leaves at different depths and heights were used to collect the depositions. A reliable analytical method for determination of azoxystrobin and tebuconazole in artificial collectors and residue samples was developed by using liquid chromatography triple-quadrupole mass spectrometry. The results showed that the atomizer distributed the droplets evenly throughout the greenhouse with good uniformity (CVs below 39%). The ratio of depositions on the internal and external sides was 66–83%, and the ratio of depositions on the underside and upper side was 39–50%. There were no significant differences in depositions between two different height crops. The residues of azoxystrobin and tebuconazole in tomato and cherry tomato fruits were far below the maximum residue limits at harvest time. In general, self-propelled high-energy ultrasonic atomizer used in a greenhouse could increase the depositions, especially on the underside and internal side of the canopies, and lead to a reduction of operator exposure risk.

## 1. Introduction

In China, the surface area covered by greenhouses to produce vegetables was around 3.92 million ha, with an annual production of 252 million tons in 2016. There are four structures of greenhouses: low tunnels, high tunnels, sunlit greenhouses, and multi-span greenhouses [1]. The sunlit greenhouse is a typical Chinese solar greenhouse mainly used in northern China. This type of greenhouse has three sides of solid walls and one side of a single slope covered by plastic films. Vegetables can be produced year-round, even in the cold regions of China. The greenhouse is characterized by moderate temperature and humidity, high planting density, and poor ventilation, while these conditions will contribute to pests and diseases spread. Thus, insecticides and fungicides are widely used in greenhouse vegetable production [2].

Tomato crops have relatively tall, wide, and dense canopies, and the passageways between rows are narrow. The tomato canopies’ characteristics and the limitations of sunlit greenhouse’s structure lead to the pest management in greenhouse challenge. However, plant protection products (PPPs) in the greenhouse of China and many other countries are normally applied by low-technology manual systems, such as knapsack sprayers and hand-held spray guns or spray booms [3,4,5,6]. These sprayers not only limit the efficiency and effectiveness of pesticide application [7], but also increase environmental contaminations, pesticide residues [8], and operator exposure risk [9,10].

In recent years, many spray technologies in greenhouses have been developed or improved in automation and intelligence (such as cold foggers [11,12], self-propelled machines [13,14]) to overcome those shortcomings. Compared to other sprayers, self-propelled machines and cold foggers can be programmed to work. They provided higher uniformity and efficiency, and more importantly, they greatly reduced operator exposure risk [15,16]. Previous studies have evaluated the spray distribution and deposition of the cold fogger. Mabbett et al. [17] has shown that the use of cold fogger can reduce the application of pesticide while maintaining the efficacy of pest and disease control. Olivet et al. [11] and Li et al. [18] found that the effective blowing distance of stationary cold fogger or hanging cold sprayer was related to the blowing speed, and the depositions reduced greatly at a greater distance from the cold fogger. Researchers proposed that with the help of auxiliary fans [19] and changing of the locations [14,20] of the cold fogger, the depositions could be increased, and they became more uniform.

In this study, cherry tomato and tomato crops with different heights in the sunlit greenhouse were chosen as the target crops, while azoxystrobin and tebuconazole were the target pesticides we used. The aim of this work was to evaluate the different height tomato crops in relation to the spray deposition and distribution, losses to the ground, and pesticide residues in the leaves, fruits, and soil, by using a newly designed self-propelled high-energy ultrasonic atomizer.

## 2. Materials and Methods

### 2.1. Spraying Equipment

**Figure 1:**
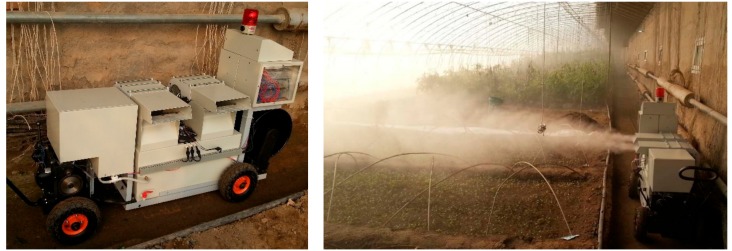
The self-propelled high-energy ultrasonic atomizer used in the tests.

The self-propelled high-energy ultrasonic atomizer (Zhong Xian Zhi Nong, Beijing, China) (Figure 1) was designed to operate automatically. The droplets were atomized by two atomizing chambers from a tank by means of ultrasonic waves, and were blown out by volute centrifugal fans. Thus, the droplets can reach the entire space of the sunlit greenhouse. The atomizer was tested with a travelling speed of 3 m/s, average air velocity of 8.7 m/s (measured by a Testo 445 anemometer with a hot ball probe, Testo, Lenzkirch, Germany), droplet volume median diameter (VMD) of 16–30 μm (measured by a Malvern Mastersizer 2000 laser particle size analyzer, Malvern, UK) and sprinkling range of 5.5–6.5 m (measured by water sensitive paper, Syngenta, Switzerland), and the application volume rate of 90 L/ha.

### 2.2. Experimental Design

The tests were carried out in a sunlit greenhouse of 667 m^2^. The tomato and cherry tomato crops we used were planted in twin rows with a crop height of 110 cm and 160 cm, respectively. Each plot grown with tomato and cherry tomato crops had a size of 7.5 m × 6.4 m, with a planting pattern of 0.65 m between plants in a row, and 0.55 m between twin plants.

**Figure 2:**
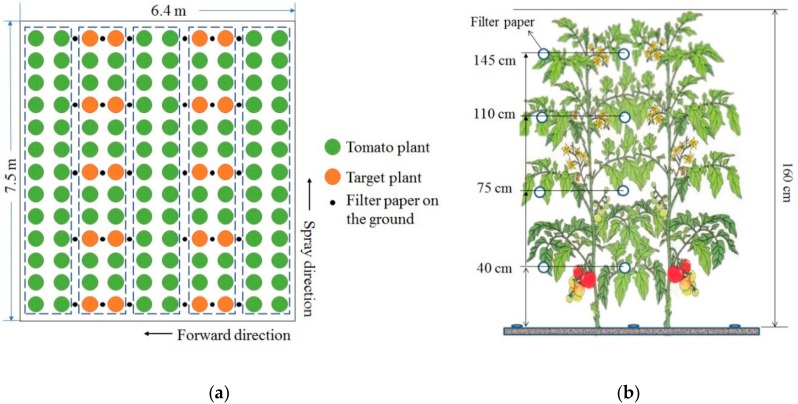
(**a**) The experimental layout for each treatment; (**b**) position of the filter papers within the crop and on the ground.

Five rows (Figure 2) were assigned for the spraying tests, of two treatments. A buffer zone was used to separate the plots from different treatments. To compare the treatments, ten pairs of plants in each treatment plot were assigned at the distances of 0.2, 2.0, 3.8, 5.6, and 7.4 m away from the atomizer. The target plants were attached with artificial collectors consisting of filter paper pieces with the size of 38.5 cm^2^ (diameter 7 cm) to simulate leaves to collect foliar spray droplets within the canopies. Each filter paper was placed over a leaf on the upper side and underside. Over each pair of tomato plants, the collectors were attached in nine zones: three heights (100 cm, 65 cm, and 30 cm) and three depths (left side, internal, and right side). Over each pair of cherry tomato plants, the collectors were attached in twelve zones: four heights (145 cm, 110 cm, 75 cm, and 40 cm) and three depths (left side, internal side, and right side). In addition, collectors were positioned on the ground coinciding with the three depths and five distances.

The solution prepared with the commercial formulations of azoxystrobin (Amistar 250 g/L SC; Syngenta China Co., Ltd., Shanghai, China) and tebuconazole (Horizon 430 g/L SC; Bayer China Co., Ltd., Beijing, China) were applied for treatments T1 and T2 (Table 1). All solutions were prepared according to the reference (normally used by farmers in the area) without any additives.

### 2.3. Sample Collection

After spraying, the filter paper samples were collected and placed in zip-lock plastic bags (85 × 120 × 0.05 mm^3^) individually. According to the standard operating procedures on pesticide registration residue field trials [21], representative soil samples, and tomato and cherry tomato fruit and leaf samples, were collected at 1, 2, 3, 5, and 7 days after spraying, to detect the residues. Samples were randomly collected at 12 sites from each plot. The soil samples were collected by a soil auger with a depth of 0–10 cm. Tomato and cherry tomato leaf and fruit samples were cut into small pieces and homogenized. Three replicates were carried out and another untreated plot was set up as a control, all samples were stored in a −20 °C freezer.

### 2.4. Analytical Methods

#### 2.4.1. Sample Preparation

Soil samples and cherry tomato and tomato leaf and fruit samples were taken from fields and stored at −20 °C until analysis. Blank samples were used for validation studies and matrix-matched standard calibrations.

The filter paper samples were extracted with 10 mL acetonitrile in the zip-lock bag. One milliliter of the solution was filtered through a 0.22 μm syringe filter, and placed into an autosampler vial for LC-MS/MS analysis.

For the analysis of soil samples, and the tomato and cherry tomato fruit and leaf samples, 10 g of homogenized samples were weighed into a 50 mL centrifuge tube. After the addition of 10 mL acetonitrile, the mixture was vigorously shaken for 3 min with a vortex mixer. Then, 3 g NaCl was added, and the sample was vortexed for 1 min and then centrifuged for 5 min at 3800 rpm. About 1 mL of the upper layer of the prepared sample was transferred into a 2 mL microcentrifuge vial containing 150 mg MgSO_4_ and 5 mg multiwalled carbon nanotubes, and then the mixture was vortexed for 30 s and centrifuged at 10,000 rpm for 1 min. Finally, the upper extract was filtered through a 0.22 μm syringe filter, and placed into an autosampler vial for LC-MS/MS analysis.

#### 2.4.2. Apparatus and LC-MS/MS Analytical Conditions

The chromatographic system was a Dionex Ultimate 3000 HPLC (Dionex, CA, USA) equipped with a reversed-phase column (Thermo Syncronis C18, 2.1 mm × 100 mm, 1.7 μm, Thermo Fisher Scientific, CA, USA). The temperature of the column was kept at 30 °C. The injection volume was 5 μL. The mobile phase was composed of acetonitrile and 0.1% aqueous formic acid (70/30, *v*/*v*) at a flow rate of 0.3 mL/min. 

Analysis of azoxystrobin and tebuconazole was carried out on a Thermo Scientific TSQ Quantum Ultra triple-quadrupole mass spectrometer (Thermo Fisher Scientific, CA, USA) using the selective reaction monitoring (SRM) mode and positive electron spray ionization (ESI+) mode. The pressure of nebulizer gas was 3.5 kV, vaporizer temperature was 300 °C, and the capillary temperature was 300 °C. The pressure of sheath gas and auxiliary gas was 35 arb and 10 arb, respectively. The collision gas pressure was 1.2 mTorr. The ion transition monitored were *m/z* 404.1 → 372.2 (quantitative) and *m/z* 404.1 → 344.2 (qualitative) for azoxystrobin, *m/z* 307.0 → 70.1 (quantitative) and *m/z* 307.9 → 150.9 (qualitative) for tebuconazole. The retention time of azoxystrobin and tebuconazole, was 1.56 min and 1.92 min, respectively.

### 2.5. Statistical Analysis

Xcalibur software with LC Quan software was used to process quantitative data obtained from the matrix-matched standard calibrations and samples. The normalized concentrations of each collector were analyzed with a factorial analysis of variance (ANOVA), and the significance of differences was evaluated by Duncan’s test for a significance level of 95%. All the analyses were made with the statistical software SPSS v.20.0 (SPSS Inc., Chicago, IL, USA).

## 3. Results and Discussion

### 3.1. Validation of Analytical Method

Linearity was studied in the range of 0.01–0.5 mg/L for azoxystrobin and tebuconazole with five calibration points (0.01, 0.05, 0.1, 0.2, and 0.5 mg/L) by matrix-matched standard calibration in blank extracts of tomato fruit, tomato leaf, soil, and filter paper samples. The calibration curves showed good linearity with correlation coefficients (*R*^2^) in the range from 0.9982 to 0.9999.

This study was performed at three fortification concentrations of 0.05, 0.1, and 0.5 mg/kg for blank samples, with an appropriate volume of working solutions. Three samples of each concentration were processed (Table 2). Average recoveries were 96–109% (tomato fruit), 88–106% (tomato leaf), 96–103% (soil), and 92–108% (filter paper), with the relative standard deviations (RSDs) less than 10%, which met the requirements of the validation criteria for pesticide residue analysis (recovery, 70–120%, RSDs ≤ 20%) proposed by EU guidelines [22]. The limit of quantifications (LOQs) of azoxystrobin and tebuconazole were established at the lowest fortified level in each matrix with the S/N ratio of 10. They ranged from 0.8 to 1.1, and from 1.1 to 1.3 μg/kg, respectively.

### 3.2. Depositon on the Canopy

Table 3 shows the results of average depositions per unit filter paper and the coefficients of variation (CVs, %) in different height crop canopies. The average depositions achieved by the self-propelled high-energy ultrasonic atomizer did not differ significantly for the two heights of tested crops. A detailed analysis of the canopy depositions showed that the depositions at different height crops were found to be in the order: cherry tomato, 30 cm > 75 cm > 110 cm > 145 cm, with significant differences between top and bottom canopies; tomato, 65 cm ≥ 30 cm > 100 cm.

In terms of the distribution uniformity, the CVs showed that the ultrasonic atomizer provided good uniformity (below 39%). This could be attributed to the mobility of the atomizer and the size of droplets (VMD of 16–30 μm). Olivet et al. [11] suggested that a greater uniformity of deposition can be achieved by smaller droplets and positioning the atomizer at different locations in the greenhouse. For tomato crops, the CVs were better (16–18%) than the cherry tomato crops (30–39%). The structure of canopies influenced the spray penetration, and the cherry tomato canopies were thicker and denser.

**Figure 3:**
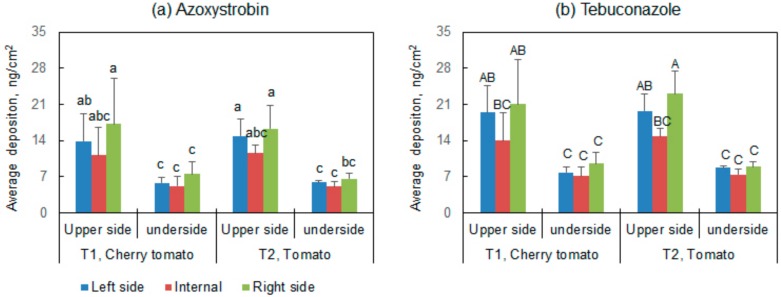
Average depositions of (**a**) azoxystrobin and (**b**) tebuconazole per unit filter paper on the left side, internal, and right side of the upper side and underside leaves (bars with different letters are significantly, Duncan’s test, *p* < 0.05).

Figure 3 shows the depositions on the upper side and underside at external and internal of canopies. The ratio between deposition on the internal and external sides was 66% to 83%. The results were greater than those obtained by spray gun or manually pulled trolley [23,24]. The depositions on the upper side were 1.0 to 1.6 times more than the depositions on the underside of the leaf. These values were similar to the results obtained by autonomous walking greenhouse mist machine [14] and greater than the results deposited by cold fogger [11] and manually pulled trolley [15].

### 3.3. Losses to the Ground

The statistical analysis showed that there were no significant differences in the values of the losses to the ground (Table 3) with different treatments. In the application with self-propelled high-energy ultrasonic atomizer, the losses to the ground were high. The mean quantity of 43.1–44.4 ng/cm^2^ of azoxystrobin and 54.7–55.3 ng/cm^2^ of tebuconazole was calculated in the ground collectors with CVs of 23–44%. The droplets distributed evenly throughout the greenhouse by airflow and deposited to the ground without the shielding of canopy. The depositions on the ground were important source of contamination. It was better to use plastic film or other things to cover the places without vegetation.

**Figure 4:**
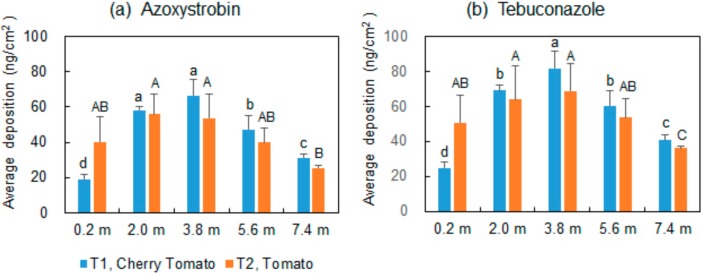
Average depositions of (**a**) azoxystrobin and (**b**) tebuconazole loss to ground at different distance away from the atomizer (bars with different letters are significantly, Duncan’s test, *p* < 0.05).

Figure 4 shows the average depositions loss to the ground in the middle were higher (*p* < 0.05) than both the sides (one side was near the plastic film, and the other was close to the solid wall). The results could be explained by the airflow field [12,25]. The droplets were redelivered by the refluent airflow by the solid wall and the plastic film.

### 3.4. Pesticide Residues in Crops and Soil

**Figure 5:**
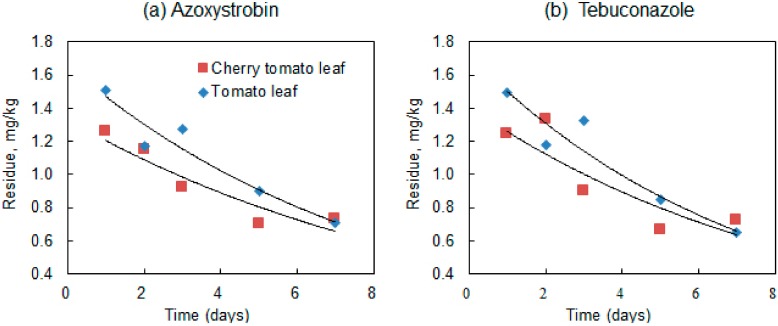
Dissipation of (**a**) azoxystrobin and (**b**) tebuconazole residue in cherry tomato and tomato leaves.

The dissipation curves of azoxystrobin and tebuconazole in cherry tomato and tomato leaves were fitted with first-order kinetics [26], as shown in Table 4 and Figure 5. As expected, a gradual and continuous decrease of fungicide residues in leaves was observed. The half-lives of azoxystrobin and tebuconazole were in the range of 5.8–6.9 days and 5.1–6.1 days, respectively. As protected environments provide defense against UV, rain, and wind, pesticide residues on crops persist for longer in protected environments than in open fields [8].

Blank samples were collected before each treatment. However, the residues of azoxystrobin in the blank soil samples were very high, and they produced unusual and unexpected results. The residues of tebuconazole in soil samples were 0.012–0.022 mg/kg. The residues of azoxystrobin in cherry tomato and tomato fruits were 0.0075–0.015 mg/kg and 0.0064–0.016 mg/kg, respectively. The residues of tebuconazole in cherry tomato and tomato fruits were 0.010–0.017 mg/kg and 0.0085–0.016 mg/kg, respectively. The residues in cherry tomato and tomato fruits were far below the maximum residue limits (MRLs) of azoxystrobin recommended by Chinese government (3 mg/kg), EU (3 mg/kg), the US government (0.2 mg/kg), and the MRLs of tebuconazole recommended by CAC (0.7 mg/kg), EU (0.9 mg/kg), and the US government (1.3 mg/kg).

## 4. Conclusions

In conclusion, the use of self-propelled high-energy ultrasonic atomizer for the application of azoxystrobin and tebuconazole on cherry tomato and tomato crops was found to distribute the droplets evenly throughout the greenhouse space. There were no significant differences in between depositions on different height crops. The canopy height and density had little effect on the uniformity. Better uniformity was found on tomato crops than cherry tomato crops and more depositions were found on the lower canopy. The ratio of depositions on the internal and external sides was between 66% to 83%, and the ratio of depositions on the underside and upper side was between 39% to 50%; they were equal to or higher than the references [11,14,23,24]. Therefore, the pests and diseases that infest the internal and underside of the leaves can be prevented or controlled better with pesticides.

The residues of azoxystrobin and tebuconazole in tomato and cherry tomato fruits were far below the MRLs set by the Chinese government, CAC, EU or the US government. Compared to conventional methods used in greenhouses, the use of self-propelled high-energy ultrasonic atomizer for the application of pesticide can be operated automatically. In general, the azoxystrobin and tebuconazole sprayed by self-propelled high-energy ultrasonic atomizer under recommended dosage would not carry potential risk to human health or food safety. This is a promising technique for applying plant protection products in a safe and efficient way in the greenhouse for crops with high, dense canopies. We plan to evaluate the application of more pesticides with the atomizer on more crops. We hope that this atomizer will be widely used in the future.

## Figures and Tables

**Table 1:** Spray conditions for the two treatments during experiments.

Treatment	Plant	Fungicide	Concentration/g a.i./L
T1	Cherry tomato	azoxystrobin	0.500
		tebuconazole	0.516
T2	Tomato	azoxystrobin	0.500
		tebuconazole	0.516

**Table 2:** Average recoveries (%), relative standard deviations (RSDs, %), and limit of quantifications (LOQs) of azoxystrobin and tebuconazole at varying fortification concentrations in the test matrices (*n* = 3).

	Average Recoveries, % (RSDs, %)			LOQ (μg/kg)
	0.05 mg/kg	0.1 mg/kg	0.5 mg/kg	
**Azoxystrobin**				
Tomato fruit	100 (6.7)	109 (1.5)	96 (4.9)	0.9
Tomato leaf	106 (4.8)	102 (2.9)	106 (4.4)	1.1
Soil	98 (7.1)	99 (7.3)	103 (3.3)	0.9
Filter paper	99 (1.6)	96 (3.2)	92 (2.8)	0.8
**Tebuconazole**				
Tomato fruit	103 (0.9)	99 (1.9)	98 (2.4)	1.2
Tomato leaf	95 (1.8)	93 (1.9)	88 (1.9)	1.3
Soil	102 (3.4)	96 (2.2)	99 (3.4)	1.2
Filter paper	107 (3.2)	108 (2.0)	107 (1.5)	1.1

**Table 3:** Average depositions and coefficients of variation for deposition per unit filter paper in the canopy and losses to the ground.

Height (cm)	Average Deposition in Canopy, ng/cm^2^ (CV, %) ^a^		Losses to the Ground, ng/cm^2^ (CV, %) ^a^	
	Azoxystrobin	Tebuconazole	Azoxystrobin	Tebuconazole
**T1, Cherry Tomato**				
145	13.0 (18) c	18.4 (19) c	44.4 (44) a	55.3 (41) a
110	15.4 (14) bc	22.1 (14) bc		
75	22.9 (35) ab	29.3 (28) ab		
30	30.5 (19) a	36.4 (15) a		
Avg.	20.3 (39)	26.6 (30)		
**T2, Tomato**				
100	15.9 (11) bc	22.5 (13) bc	43.1 (29) a	54.7 (23) a
65	22.5 (23) ab	30.7 (21) ab		
30	22.1 (13) ab	29.5 (22) ab		
Avg.	20.2 (18)	27.6 (16)		

^a^ Average deposition in the same column with different letter represent significantly differences (*p* < 0.05, Duncan’s test).

**Table 4:** Half-life and other parameters for azoxystrobin and tebuconazole dissipated in leaves.

Matrix	Fungicide	Regression Equation	Determination Coefficient (R^2^)	Half-Life (Days)
Cherry tomato leaves	Azoxystrobin	C_t_ = 1.3329e^−0.101t^	0.8615	6.9
	Tebuconazole	C_t_ = 1.4117e^−0.114t^	0.7691	6.1
Tomato leaves	Azoxystrobin	C_t_ = 1.6581e^−0.12t^	0.9413	5.8
	Tebuconazole	C_t_ = 1.7237e^−0.137t^	0.9299	5.1

C represents the concentration of azoxystrobin or tebuconazole residue at time t.
